# CT-Defined Low Skeletal Muscle Mass, Prognostic Nutritional Index, and Anemia in Relation to In-Hospital Major Morbidity After Gastrectomy for Gastric Carcinoma: A Retrospective Cohort Study

**DOI:** 10.3390/medicina62101949

**Published:** 2026-10-09

**Authors:** Mujdat Turan, Sumeyra Guler, Yasin Hatipoglu, Ibrahim Burak Bahcecioglu, Mehmet Ali Gulcelik

**Affiliations:** 1 Department of General Surgery, Gulhane Faculty of Medicine, Ankara Gulhane Research and Training Hospital, Health Sciences University, 06010 Ankara, Turkey; yasinhatipoglu446@hotmail.com; 2 Department of Surgical Oncology, Gulhane Faculty of Medicine, Ankara Gulhane Research and Training Hospital, Health Sciences University, 06010 Ankara, Turkey; guler.sumeyra@yahoo.com (S.G.); dr.ibb@hotmail.com (I.B.B.); mehmetali.gulcelik@sbu.edu.tr (M.A.G.)

**Keywords:** gastrectomy, low skeletal muscle mass, prognostic nutritional index, anemia, postoperative complications, in-hospital major morbidity

## Abstract

*Background and Objectives*: Computed tomography (CT)-defined skeletal muscle mass, nutritional status, and anemia are widely proposed markers of perioperative vulnerability before gastrectomy, but published estimates are heterogeneous and derive largely from East Asian, upfront-surgery cohorts. It is unclear how frequently these abnormalities occur in a predominantly neoadjuvant-treated cohort, and how precisely a single-center series can relate them to severe early morbidity. *Materials and Methods*: This retrospective, single-center observational cohort included 107 consecutive patients who underwent gastrectomy for histologically confirmed gastric carcinoma. The outcome was an in-hospital Clavien–Dindo grade ≥III complication. Exposure prevalence is reported with Wilson 95% confidence intervals (CIs). Exploratory unadjusted logistic regression was used to estimate odds ratios (ORs), and Firth bias-reduced logistic regression was used as a sparse-event sensitivity analysis. Sensitivity analysis applied gastric-cancer-specific muscle thresholds and excluded completion gastrectomies. *Results*: CT-defined low skeletal muscle mass, low Prognostic Nutritional Index (PNI < 46), and anemia were present in 67 (62.6%; 95% CI 53.2–71.2), 45 (42.1%; 33.1–51.5), and 71 (66.4%; 57.0–74.6) patients, respectively. Fifteen patients (14.0%; 95% CI 8.7–21.8) had major complications, of whom 14 (93.3%) had undergone open surgery. All estimates were unadjusted and imprecise, with confidence intervals spanning potentially clinically important associations in both directions: low skeletal muscle mass (OR 1.23, 95% CI 0.39–3.89), SMI per 5-cm^2^/m^2^ decrease (OR 1.20, 0.88–1.65), PNI per 5-unit decrease (OR 1.18, 0.80–1.75), low PNI (OR 1.24, 0.42–3.72), and anemia (OR 1.02, 0.32–3.23). Firth penalized estimates were materially similar to the conventional estimates. Substituting gastric-cancer-specific muscle thresholds lowered the prevalence of low muscle mass from 62.6% to 21.5% without resolving the estimate (OR 1.40, 0.40–4.88). Excluding the 20 completion gastrectomies (87 patients, 11 events) moved the low-muscle-mass estimate below unity (OR 0.74, 0.21–2.65) without improving precision. *Conclusions*: These abnormalities were highly prevalent, but with only 15 events, the confidence intervals did not exclude clinically important associations in either direction. The concentration of major events among patients undergoing open surgery underscores the potential influence of operative case mix and residual confounding. The results support neither causal nor independent-effect inference whether assessment of these parameters adds to perioperative risk stratification remains a hypothesis to be tested in larger, multicenter, well-characterized cohorts.

## 1. Introduction

Gastric cancer remains a major global malignancy and a substantial cause of cancer mortality [1,2]. Surgery for gastric malignancy has a long trajectory: the first successful distal gastric resections performed by Theodor Billroth in the early 1880s established that the stomach could be resected and reconstructed with survivable risk, and the reconstructions bearing his name remain in routine use nearly a century and a half later [3]. What has changed is the context in which the operation is performed. For resectable gastric carcinoma, gastrectomy with appropriate lymphadenectomy remains central to curative-intent multidisciplinary care, but it is now embedded in a multimodal pathway in which most patients in Western practice receive perioperative chemotherapy before resection [2,4]. Postoperative morbidity remains clinically important because it may prolong hospitalization, increase care requirements, and delay oncologic treatment [5]. The Clavien–Dindo classification grades complications according to the treatment required; grade III or higher events represent morbidity requiring invasive intervention, intensive care, or life-sustaining treatment [6].

Contemporary sarcopenia definitions distinguish muscle strength, muscle quantity or quality, and physical performance; low muscle strength is central to diagnosis, whereas reduced muscle quantity confirms the diagnosis [7]. A single L3 CT-derived skeletal muscle index (SMI) measures muscle quantity but does not by itself establish this multidimensional syndrome. This distinction is not semantic. A patient with preserved strength and a low SMI and a patient with clinically evident functional decline carry different perioperative risk, yet both are frequently labeled “sarcopenic” in the surgical literature, which is one reason published estimates are difficult to reconcile. Accordingly, the present study uses the term “CT-defined low skeletal muscle mass.” Reported associations between CT body composition and postoperative outcome in gastric cancer are correspondingly inconsistent, ranging from a substantially increased pooled risk of major complications in meta-analyses to no detectable association in cohorts that use sex- and body-mass-index-specific thresholds [8,9,10,11,12,13,14]. This inconsistency is not random. It tracks systematic between-study differences in source population, scan timing, segmentation protocol, choice of cutoff, inclusion of functional testing, endpoint definition, and adjustment strategy, and it means that series are comparable only to the extent that each of these elements is reported explicitly.

The Prognostic Nutritional Index (PNI), calculated from serum albumin and lymphocyte count, is an accessible nutritional–immune marker that has been evaluated in gastric cancer surgery [15,16]. Preoperative anemia is also common in gastrectomy populations, but binary hemoglobin thresholds do not describe its severity, cause, iron status, bleeding, treatment, or relationship to transfusion [17,18]. Three gaps follow from this. First, the available CT body-composition evidence in gastric cancer is derived predominantly from East Asian cohorts treated largely via upfront surgery, whereas contemporary Western and Turkish practice is dominated by perioperative chemotherapy; how frequently low muscle mass, impaired nutritional–immune status, and anemia occur in such a population has rarely been quantified with explicit interval estimates. Second, in small single-center cohorts with few major morbidity events, multivariable estimation may be unstable and vulnerable to sparse-data bias, making it difficult to distinguish exposure effects from an operative and clinical case mixture [19,20,21]. Third, the operative question is not whether an association exists in principle but how far a single institution’s data can legitimately inform a preoperative risk discussion. The present study addresses these gaps by reporting exposure prevalence with interval estimates, quantifying the dependence of the muscle findings on the choice of threshold, and declining to present unadjusted estimates as independent predictors.

This retrospective cohort study therefore explored the separate associations of preoperative CT-defined low skeletal muscle mass, continuous SMI, continuous PNI, low PNI, and anemia with in-hospital Clavien–Dindo grade ≥III morbidity after gastrectomy for gastric carcinoma. The secondary objective was to characterize the prevalence of these three preoperative abnormalities in a consecutive, predominantly neoadjuvant-treated Turkish gastrectomy cohort. It was not designed to estimate causal effects, develop a prediction model, or establish a risk score.

## 2. Materials and Methods

### 2.1. Study Design

This retrospective, single-center observational cohort study was reported in accordance with STROBE [22]. Consecutive patients who underwent gastrectomy for a histologically confirmed primary gastric carcinoma between 30 November 2023 and 1 February 2026 were reviewed. The patient was the unit of analysis. The study was not designed or analyzed as a clinical prediction model, risk score, or assessment of independent predictive performance. The Ankara Gülhane Research and Training Hospital Ethics Committee approved the study (E-50687469-799-308105246; 31 March 2026). Because the study used retrospectively collected hospital-archive data and institutional authorization for retrospective data use had been obtained, the Ethics Committee determined that separate informed consent for research participation was not required. Routine surgical consent was not treated as research consent.

### 2.2. Data Source, Patient Flow, and Study Population

**Figure 1:**
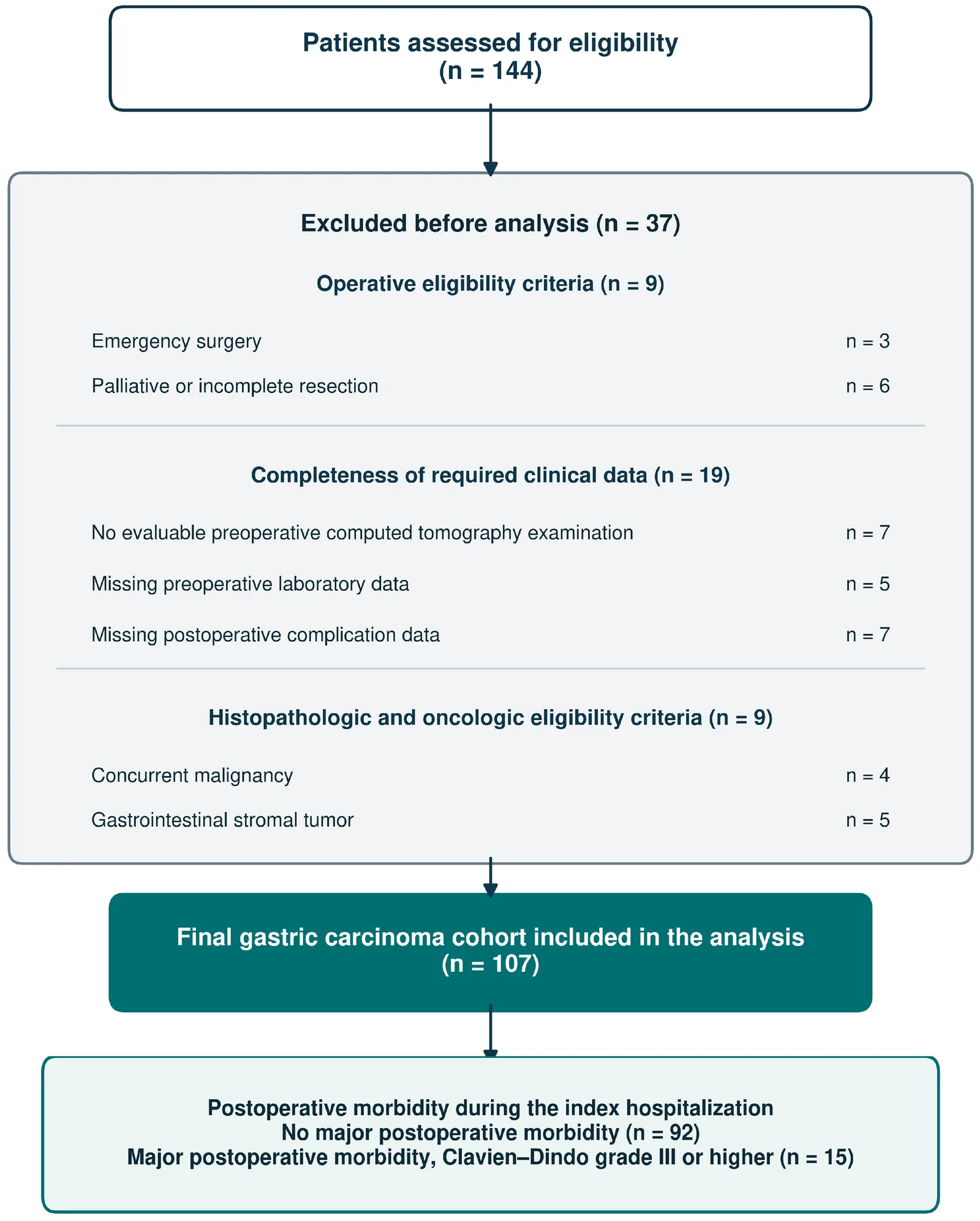
STROBE-style participant flow. Of 144 patients assessed for eligibility, 37 were excluded before final analysis: nine for operative eligibility criteria, 19 for incomplete clinical data, and nine for histopathologic or oncologic eligibility criteria. The final gastric-carcinoma cohort comprised 107 patients; 92 had no major postoperative morbidity and 15 experienced in-hospital Clavien–Dindo grade III or higher morbidity.

Data were obtained retrospectively from institutional medical records, operative records, pathology reports, laboratory databases, and preoperative imaging records. A total of 144 patients were assessed for eligibility. Histological diagnosis was taken from the definitive resection pathology report in every case. Thirty-seven patients were excluded because of emergency surgery (*n* = 3), palliative or incomplete resection (*n* = 6), no evaluable preoperative CT examination (*n* = 7), missing preoperative laboratory data (*n* = 5), missing postoperative complication data (*n* = 7), concurrent malignancy (*n* = 4), or a definitive histological diagnosis of gastrointestinal stromal tumor rather than gastric carcinoma (*n* = 5). The final gastric carcinoma cohort comprised 107 patients (Figure 1).

Eligible patients underwent total, distal, proximal, or completion gastrectomy for a histologically confirmed primary gastric carcinoma and had the CT, hemoglobin, albumin, lymphocyte, and in-hospital complication data required for the analyses. Twenty completion gastrectomies were retained in the carcinoma cohort; these procedures followed prior antrectomy for benign peptic-ulcer perforation rather than prior oncologic gastric resection. Completion gastrectomy therefore accounted for 20 of 107 procedures (18.7%), a higher proportion than in most contemporary series and a reflection of the historical frequency of antrectomy for benign peptic-ulcer disease in this referral population. These patients met every eligibility criterion and were retained because excluding them would have removed a clinically relevant and technically demanding subgroup; the additional case-mix heterogeneity and the adhesion-related operative difficulty that such procedures entail are acknowledged among the limitations, and their influence on the principal estimates was examined in a sensitivity analysis that excluded them (Section 2.5).

Exclusion criteria were emergency surgery, palliative or incomplete resection, prior gastric resection for malignancy, concurrent active malignancy requiring treatment, unsuitable preoperative CT, missing preoperative laboratory data, missing complication outcome data, or histology other than gastric carcinoma. No data were missing for the analysis variables among the 107 included patients; analyses were complete-case, and no imputation was performed.

### 2.3. Clinical, Operative, Imaging, and Laboratory Measurements

Recorded variables included age, sex, body mass index (BMI), American Society of Anesthesiologists (ASA) physical status, neoadjuvant therapy, surgical approach, gastrectomy type, pathological T and N categories, and histological type. ASA was summarized as I–II versus III–IV [23]. The surgical approach was open or laparoscopic; gastrectomy was distal, proximal, total, or completion. D2 lymphadenectomy was the institutional approach for carcinoma resections; the total number of lymph nodes retrieved was recorded and is reported as the only available surrogate of dissection extent. No multivisceral resection or conversion occurred, and all resections were recorded as R0. Operative duration, blood loss, intraoperative transfusion, reconstruction method, tumor location, surgeon volume, and perioperative care-pathway variables were not available in retrievable form in the institutional archive for the full study period; their absence is treated as a principal limitation rather than an incidental one.

Skeletal muscle was manually segmented on a single 1 mm axial CT slice at the third lumbar vertebral level with CoreSlicer 1.0, using a −29 to +150 Hounsfield unit threshold [24,25]. The SMI was calculated as skeletal muscle area divided by height squared (cm^2^/m^2^). In patients who received neoadjuvant treatment, the scan used for operability assessment was obtained after treatment, approximately one month before surgery; in patients without neoadjuvant treatment, the scan was obtained within three months before surgery. Contrast phase was not recorded. One unblinded observer performed all measurements; observer experience was not documented, and no intraobserver or interobserver reliability assessment was available. Duplicate segmentations were not retained in the study database, so a reliability substudy could not be reconstructed retrospectively; this is treated as a primary rather than a general limitation, and no claim of measurement reproducibility is made anywhere in this manuscript.

The literature-derived Prado thresholds (<52.4 cm^2^/m^2^ for men and <38.5 cm^2^/m^2^ for women) defined a low SMI [26]. These cutoffs originated in an obese mixed respiratory and gastrointestinal solid-tumor cohort and were not developed for a Turkish gastrectomy population. Because both exposure prevalence and the resulting estimates are sensitive to this choice, every analysis involving dichotomized muscle mass was repeated using the gastric-cancer-specific thresholds of Zhuang et al. (<40.8 cm^2^/m^2^ for men and <34.9 cm^2^/m^2^ for women), derived in a large-scale gastrectomy cohort with CT performed within 30 days of surgery [11]. Because muscle strength and physical performance were unavailable, CT-defined low skeletal muscle mass was not interpreted as functionally defined sarcopenia. Continuous SMI, scaled per 5-cm^2^/m^2^ decrease, was used to reduce dependence on a single dichotomization.

The laboratory value closest to surgery, within seven days preoperatively, was used. The PNI was calculated as 10 × serum albumin (g/dL) + 0.005 × total lymphocyte count (/mm^3^) [15]. The PNI was computed directly from the recorded serum albumin and absolute lymphocyte count for every patient, and this computed value was used in all analyses. A separately entered PNI field present in the source database was not used because it did not reproduce the formula in every record. The continuous PNI, scaled per 5-unit decrease, was the primary PNI parameter. A PNI <46 was retained as a secondary, literature-referenced dichotomization because this threshold had been derived in a prior gastric cancer total-gastrectomy cohort [16]; it was not prespecified for the present cohort, and no data-driven cutoff was sought. The source files did not provide patient-level timing, dose, or indication data for preoperative red-cell transfusion, albumin replacement, iron therapy, or nutritional intervention. These preoperative interventions were not used to define the postoperative Clavien–Dindo grade ≥III study outcome. Preoperative C-reactive protein was also recorded and is reported descriptively; it was not analyzed as an additional exposure, because a fourth exposure in a dataset containing 15 events would compound multiplicity without adding precision.

Anemia was defined using the 2024 World Health Organization sex-specific thresholds for adults aged 15–65 years (<13 g/dL for men and <12 g/dL for nonpregnant women) [18]. These thresholds were also applied to patients older than 65 years because the source dataset contained no alternative prespecified definition for that age group; this is a classification limitation. The dataset did not contain anemia severity, etiology, iron indices, chronic-disease classification, bleeding status, chemotherapy attribution, or sufficiently detailed transfusion data. Pathological T and N variables were recorded according to the American Joint Committee on Cancer Staging Manual, eighth edition, and were summarized as T3–T4 versus lower categories and N-positive versus N0 [27]. Among the 86 patients who received neoadjuvant therapy, these were considered post-treatment categories; they were tabulated together with the primary pathological categories for the 21 patients treated by upfront surgery. The combined variable should therefore be read as a descriptor of resected-specimen tumor burden rather than of baseline clinical stage.

### 2.4. Outcome Definition

The outcome was any postoperative complication of Clavien–Dindo grade III or higher during the index hospitalization [6]. Treating clinicians assigned grades according to the Clavien–Dindo framework. When a patient had multiple complications, the highest grade was retained. Events identified after discharge, during readmission, or at another hospital were not captured. The available data did not document the number or experience of graders or blinding to the exposures. Two features of this definition constrain interpretation and are therefore stated here rather than only among the limitations. First, grading was performed by the treating teams in the course of routine clinical care and not by a designated adjudicator blinded to exposure status, so exposure-related differential misclassification cannot be excluded. For example, if patients with visibly poor nutritional status were monitored more intensively, their complications would be more completely captured. Second, outcome ascertainment ended at index discharge, so the endpoint is in-hospital major morbidity and is narrower than the 30-day or 90-day endpoints used in much of the comparative literature.

### 2.5. Statistical Analysis

The analysis-ready file was maintained in IBM SPSS Statistics for Windows, version 27.0 (IBM Corp., Armonk, NY, USA). Descriptive summaries and conventional logistic regressions were independently reproduced in R version 4.6.0 using haven version 2.5.5, and Firth sensitivity analyses were independently verified using logistf version 1.26.1. Continuous variables are reported as mean ± standard deviation or median [interquartile range], according to distribution; categorical variables are reported as *n* (%). Table 1 is descriptive and contains no baseline significance tests. Prevalence estimates for the binary exposures and the outcome are reported with Wilson 95% CIs because the Wilson interval retains nominal coverage at the sample size and available event count.

Separate unadjusted binary logistic regressions estimated odds ratios (ORs), Wald 95% confidence intervals (CIs), and two-sided Wald *p* values for CT-defined low skeletal muscle mass, SMI per 5-cm^2^/m^2^ decrease, PNI per 5-unit decrease, low PNI, and anemia. For regression coding, the continuous exposure terms were defined as −SMI/5 and −PNI/5 so that the corresponding ORs represent each 5-unit decrease. Because only 15 outcome events occurred, no multivariable model could adequately address the clinically relevant confounding structure without substantial overfitting and sparse-data bias. Age- and sex-adjusted estimates are therefore not reported, since they could not legitimately be described as clinically adjusted or independent. Firth bias-reduced logistic regression was used as a sensitivity analysis for each one-exposure model. Firth estimates were obtained with Jeffreys-prior penalization; 95% CIs were based on the profile penalized likelihood, and two-sided *p* values were obtained via penalized likelihood-ratio tests [19,21].

The OR was the sole regression effect measure. Modified Poisson risk-ratio models, the four-category joint-exposure model and multiplicative interaction analysis were not included because they would have added analytical complexity without reliable precision at the observed event count. No post hoc power calculation or data-derived cutoff was used. The fixed retrospective sample and 15 events were evaluated through effect estimates, CI width, and model stability. All *p* values were two-sided; *p* < 0.05 was used only as a conventional reference and not as a dichotomous criterion for clinical relevance. In accordance with current guidance on the reporting of sparse-data analyses, interpretation throughout this manuscript rests on the point estimate and the full width of its confidence interval rather than on the associated test statistic [20]. Wald and penalized likelihood-ratio *p* values are retained in Table 2 for completeness. All dichotomous muscle-mass analyses were repeated using the gastric-cancer-specific SMI thresholds of Zhuang et al. [11] in place of the Prado thresholds [26], to quantify the dependence of the findings on a single dichotomization. In a second sensitivity analysis, the 20 completion gastrectomies were excluded and all five one-exposure models were re-estimated with both conventional and Firth regression, because these procedures were performed in previously operated abdomens and constitute a technically distinct subgroup whose case mix could not otherwise be addressed. Both analyses were exploratory and was not used to select a preferred estimate; both are reported in full. Because the gastrointestinal stromal tumor lies outside of the target population, reinstatement of the five records excluded on definitive histology was not regarded as a test of clinically meaningful uncertainty; it is reported only in Supplementary Table S1, solely to document that this post hoc eligibility correction did not alter the principal estimates. A measurement-reliability substudy was not feasible because duplicate segmentations were not retained in the study database.

## 3. Results

### 3.1. Baseline Sample Characteristics

Among the 144 patients assessed for eligibility, 37 were excluded; the final gastric carcinoma cohort comprised 107 patients (Figure 1). Fifteen patients (14.0%; 95% CI 8.7–21.8) had an in-hospital Clavien–Dindo grade ≥III complication. CT-defined low skeletal muscle mass was present in 67 patients (62.6%; 95% CI 53.2–71.2), low PNI (<46) in 45 (42.1%; 33.1–51.5), and anemia in 71 (66.4%; 57.0–74.6). Each of the three preoperative abnormalities was therefore present in a substantial minority to a clear majority of the cohort, and unlike the exposure–outcome associations reported below their prevalence could be estimated with reasonable precision at this sample size. Applying the gastric-cancer-specific thresholds instead identified 23 patients (21.5%; 95% CI 14.8–30.2) as having low muscle mass. The distribution of clinical, operative, tumor, muscle, nutritional, and hematologic variables is summarized in Table 1.

The carcinoma cohort was surgically heterogeneous: 76 patients (71.0%) underwent open surgery, and gastrectomy included distal, proximal, total, and completion procedures. Open surgery occurred in 14 of 15 patients with major morbidity (93.3%), compared with 62 of 92 without major morbidity (67.4%). These differences are descriptive and were not used to infer adjusted exposure effects. They are, however, the dominant feature of the dataset: with 14 of 15 events concentrated in a single operative category, any unadjusted exposure–outcome estimate reported below is confounded by the operative approach to a degree that these data cannot quantify. Lymph node yield, the one available surrogate of dissection extent, was similar between groups, although lymph node yield is an imperfect surrogate for operative complexity (median 22 [IQR 19.0–28.0] without major morbidity versus 22 [15.5–30.0] with major morbidity).

### 3.2. Exploratory Association Estimates

All estimates reported in this section are unadjusted and exploratory; because 14 of the 15 major events followed open surgery, they remain confounded by operative approach and complexity to an extent that these data cannot quantify. All conventional and Firth estimates were accompanied by wide CIs (Table 2). For example, the conventional OR for CT-defined low skeletal muscle mass was 1.23 (95% CI 0.39–3.89), and the Firth estimate was 1.18 (95% profile CI 0.40–3.84). The lower limit of that interval corresponds to an approximately 60% reduction and the upper limit to an almost fourfold increase in the odds of major morbidity; the data are therefore equally compatible with a clinically important protective association, no association, and a clinically important harmful association. Continuous SMI and PNI estimates were also imprecise. Accordingly, the data did not distinguish clinically important lower from higher odds for any evaluated exposure, and no exposure is reported here as a predictor of major morbidity.

### 3.3. Postoperative Outcomes

Of the 15 major events, 12 were grade III, two were grade IV, and one was grade V; the grade V event was the only in-hospital death. The median length of stay was 9.0 days [IQR 7.5–12.0] overall, 8.5 days [7.0–11.0] without major morbidity, and 21.0 days [13.0–34.5] with major morbidity. The principal recorded major complications are listed in Table 3. Eight of the 15 events (53.3%) were pancreatic fistula, duodenal stump leak, or combined duodenal stump and anastomotic leak—complications for which operative and reconstructive factors are likely to be important.

### 3.4. Sensitivity Analysis

Substituting the gastric-cancer-specific thresholds of Zhuang et al. [11] for the Prado thresholds [26] reduced the prevalence of CT-defined low skeletal muscle mass from 67 patients (62.6%) to 23 (21.5%; 95% CI 14.8–30.2)—a nearly threefold change produced by the choice of cutoff alone, with no change in the underlying measurements. The two classifications were perfectly nested: all patients identified as having low muscle mass according to the Zhuang criteria were also identified as having low muscle mass by the Prado criteria, while the 44 patients who differed were those whose SMI fell between the two thresholds. The association with in-hospital major morbidity was marginally larger but no more precise (four of 23 exposed patients had an event; OR 1.40, 95% CI 0.40–4.88; Firth OR 1.47, 95% profile CI 0.40–4.67). Reducing exposure prevalence by two-thirds therefore changed neither the direction nor the resolution of the estimate. This analysis is summarized in Table 4. Excluding the 20 completion gastrectomies left 87 patients with 11 major events (12.6%; 95% CI 7.2–21.2). Major morbidity had occurred in four of the 20 completion procedures (20.0%; 95% CI 8.1–41.6), 19 of which had been performed by an open approach; two of these four events involved the duodenal stump. The completion subgroup was older than the remaining cohort (mean age 66.8 ± 12.6 versus 61.4 ± 10.9 years), and all four of its events occurred in patients classified as having low muscle mass by the Prado criteria. Consequently, the dichotomous low-muscle-mass estimate fell below unity after exclusion (six of 53 exposed patients had an event; OR 0.74, 95% CI 0.21–2.65; Firth OR 0.73, 95% profile CI 0.21–2.60), whereas the continuous SMI estimate was essentially unchanged (OR 1.26, 0.86–1.84; Firth OR 1.23, 0.87–1.84). Estimates for PNI per 5-unit decrease (OR 1.08, 0.67–1.75), low PNI (OR 1.28, 0.36–4.56), anemia (OR 0.81, 0.22–3.02), and low muscle mass by the Zhuang criteria (OR 1.66, 0.39–7.07) remained compatible with the principal analysis, with wider intervals (Table 4). The reversal of the dichotomous muscle estimate after the removal of four events is the most informative result of this analysis: at this event count, point estimates are governed by a handful of patients concentrated in a technically distinct subgroup, and none of the estimates should therefore be interpreted directionally. Finally, reinstating the five records excluded on histological grounds, reported for transparency only, left all five principal estimates materially unchanged (Supplementary Table S1).

## 4. Discussion

Before the individual findings are discussed, it should be stated that the principal contribution of this study is descriptive and methodological rather than inferential. In a consecutive, predominantly neoadjuvant-treated cohort, low skeletal muscle mass, low PNI, and anemia were common, whereas the limited number of major morbidity events precluded precise estimation of exposure–outcome associations or defensible multivariable adjustment. In addition, the marked change in low-muscle-mass prevalence when alternative published thresholds were applied illustrates the dependence of body-composition estimates on cutoff selection. Thus, the study contributes estimates of prevalence, cutoff sensitivity, and statistical uncertainty rather than evidence of independent prognostic effects. This retrospective, single-center gastric-carcinoma cohort yielded imprecise, unadjusted, exploratory estimates for CT-defined low skeletal muscle mass, continuous SMI, PNI, low PNI, and anemia in relation to in-hospital Clavien–Dindo grade ≥III morbidity. Only 15 events occurred; consequently, every CI was wide and allowed both clinically important lower and higher odds. The findings therefore cannot support a conclusion of absence, equivalence, causal effect, or independent predictive performance.

The muscle findings should be interpreted against substantial methodological heterogeneity in the gastric cancer surgery literature. Meta-analyses by Kamarajah et al. and Chen et al. pooled studies using different body-composition techniques, cutoffs, populations, and complication definitions and reported higher pooled major-complication risk among patients labeled as sarcopenic [8,9]. In contrast, Tegels et al. used sex- and BMI-specific CT thresholds and an in-hospital endpoint and reported no clear association with severe complications [12]. The present Prado thresholds (52.4 cm^2^/m^2^ for men and 38.5 cm^2^/m^2^ for women) were derived from an obese mixed-tumor population [26], whereas Zhuang et al. developed lower gastric-cancer-specific values (40.8 and 34.9 cm^2^/m^2^) and used CT within 30 days of surgery [11]. These differences can materially change exposure prevalence and estimated associations; a new outcome-driven cutoff was therefore not derived here, and the Zhuang thresholds were instead applied as a sensitivity analysis. That analysis quantifies the point directly. In the same 107 patients, the two published thresholds classified 62.6% and 21.5%, respectively as having low muscle mass, a difference of 44 patients arising from the cutoff alone. Reported using only the Zhuang criteria, this cohort’s prevalence would sit among the lowest figures in the literature; whereas when reported using only the Prado criteria, it is among the highest. The estimated association meanwhile changed from 1.23 (0.39–3.89) to 1.40 (0.40–4.88), which is to say that it did not change in any interpretable sense. This is a compact illustration of why prevalence figures and effect estimates from body-composition studies in gastric cancer cannot be compared across series unless the threshold, the reference population from which it was derived, and the scan timing are all stated explicitly.

Body-composition change during neoadjuvant therapy has been documented and complicates comparison across studies using pre- or post-treatment imaging [28]. Terminology is also important. Prospective gastric-cancer studies by Wang et al., Zhou et al., Fukuda et al., and Zhuang et al. incorporated handgrip strength and/or gait speed in addition to muscle quantity and reported associations with postoperative complications [10,13,14,29]. The present dataset contained neither strength nor performance, so an L3 SMI classification cannot be generalized to contemporary functionally defined sarcopenia [7]. Measurement uncertainty is also plausible because the CT window differed by neoadjuvant-treatment status, 80.4% of the carcinoma cohort received neoadjuvant therapy, only one unblinded observer segmented the images, contrast phases were unavailable, and no reliability study was possible; this last point is addressed as a primary rather than a general limitation. Recent robotic and paired DXA/CT cohorts further illustrate how operative approach and diagnostic criteria can change the observed estimates [30,31].

For the PNI, Jiang et al. derived a cutoff of 46 in 386 patients undergoing total gastrectomy and evaluated broader postoperative complications and long-term outcomes [16]. That threshold was not prespecified for the present mixed gastrectomy cohort; the continuous PNI was therefore the principal PNI parameter and the dichotomy was explicitly secondary. Neither estimate was sufficiently precise to determine the direction or magnitude of an association with the narrower in-hospital grade ≥III endpoint. Moreover, the albumin and lymphocyte count may reflect inflammation, treatment, and nutrition; the absence of patient-level timing and dose data for preoperative albumin replacement or other nutritional interventions limits the assessment of possible exposure misclassification and preoperative C-reactive protein, which was recorded as not differing appreciably between the two outcome groups.

The anemia result is similarly non-definitive. In 9936 National Surgical Quality Improvement Program gastrectomies, Kouyoumdjian et al. modeled hematocrit nonlinearly, assessed 30-day outcomes, and examined perioperative transfusion; the design, exposure, endpoint, and covariate information were substantially richer than in the present cohort [17]. A separate 2163-patient gastric-cancer cohort also used more detailed clinical adjustment than was possible here [32]. In the present study, anemia was reduced to a WHO sex-specific binary variable; the 2024 WHO adult thresholds are specified for ages 15–65 years, whereas 43 patients in the carcinoma cohort were older than 65 years. Because no alternative prespecified threshold was available, the same thresholds were retained for the whole cohort, and this age-range extrapolation is acknowledged as a classification limitation. Severity, cause, iron status, chronic disease, bleeding, chemotherapy contribution, and detailed transfusion data were unavailable. The broad CIs around the ORs therefore should not be read as evidence that preoperative hematologic status is clinically irrelevant.

Operative and tumor-related case mix also limits interpretation. Open surgery was more common among patients with major morbidity, and procedures ranged from distal to completion gastrectomy. ASA class, T and N categories, neoadjuvant therapy, and surgical approach were available, but operative duration, blood loss, transfusion, reconstruction, tumor location, surgeon experience, and care-pathway data were not. With only 15 events, incorporating even the available covariates would have produced an unstable, incompletely adjusted model. The reported estimates are therefore unadjusted and exploratory; residual confounding remains substantial. This omission is not peripheral, and it deserves more than a single clause. Major morbidity after gastrectomy is driven substantially by operative complexity and intraoperative course: prolonged operative time, greater blood loss, and intraoperative transfusion are each associated with severe complications, while reconstruction type and tumor location determine which anastomoses and which dissection planes are placed at risk. The observed distribution of complications is consistent with that logic. Pancreatic fistula, duodenal stump leak, and combined duodenal stump and anastomotic leak together accounted for eight of the 15 major events—complications that are strongly influenced by the extent and technical characteristics of dissection and reconstruction. Twenty of the 107 procedures were completion gastrectomies performed in previously operated abdomens, and 14 of the 15 events followed open surgery. The completion subgroup warrants separate comment. These operations followed prior antrectomy and were therefore performed in fields with adhesions and altered anatomy of the gastric remnant and duodenal stump; 19 of the 20 were performed by an open approach, and the event rate in this subgroup (4 of 20, 20.0%) exceeded that of the remaining procedures (11 of 87, 12.6%). Because the subgroup was also older and all four of its events occurred in patients with low muscle mass, it contributed simultaneously to the exposure and to the outcome, and its exclusion reversed the direction of the dichotomous muscle estimate without narrowing the interval. Completion gastrectomy should therefore be regarded not merely as a source of additional variance but as a plausible confounder whose influence cannot be disentangled from that of open surgery in the present data. The one operative-extent variable that could be retrieved, lymph-node yield was very similar between groups, although it is an imperfect surrogate for operative complexity and cannot exclude differences in the technical extent or difficulty of surgery. Taken together, these features describe a confounding structure that the present dataset cannot address at any level of statistical sophistication, and whose net direction cannot be predicted. Preoperative frailty may plausibly have influenced the decision to operate openly, in which case part of any true association would be absorbed into the operative variable; conversely, the more complex operations may have been performed on fitter patients. Both mechanisms are compatible with the data, which is precisely why no adjusted estimate is offered. A two-center study of combined muscle and PNI status focused on long-term prognosis in advanced gastric cancer and does not resolve the present in-hospital sparse-event question [33].

This study has identifiable strengths. The cohort comprised consecutive, unselected patients that met the stated eligibility criteria; no data were missing for any variable used in the analyses; the exposure definitions and thresholds were taken from the published literature rather than derived from these data; both a conventional and a bias-reduced estimator were applied; the dependence of the muscle findings on the choice of threshold was quantified rather than assumed; and all descriptive and regression results were independently reproduced in a second statistical environment directly from the source data file.

Four primary limitations exist, and they determine what the study can and cannot support. (i) Precision: Fifteen events cannot resolve associations of the magnitude at issue, and every confidence interval reported here spans values whose clinical implications differ materially from one another. This, rather than the absence of statistically significant findings, is the central constraint. (ii) Confounding by operative and clinical case mix: The cohort is heterogeneous in terms of surgical approach, extent of gastrectomy, age, ASA class, and tumor characteristics, and 14 of 15 major events occurred after open surgery; key perioperative variables were unavailable. Exclusion of the 20 completion gastrectomies reversed the direction of the dichotomous muscle estimate, which illustrates how strongly these estimates depend on case mix. No estimate reported here should be read as adjusted or independent. (iii) Measurement reliability: Segmentation was performed by a single unblinded observer with undocumented experience, contrast phase was not recorded, and scan timing differed systematically by neoadjuvant status. Duplicate segmentations were not retained, so no intraobserver or interobserver reliability coefficient can be reported, and the reproducibility of the muscle measurements is unverified. (iv) Outcome ascertainment: The endpoint was restricted to Clavien–Dindo grade ≥III events during the index hospitalization, graded by treating clinicians in routine care without documented blinding to exposure status and without a designated adjudicator; events after discharge, on readmission, or at another institution were not captured. Non-differential misclassification would tend to attenuate estimates, but differential misclassification cannot be excluded. Secondary limitations include the retrospective single-center design with its attendant selection and information bias; exclusion of 37 of 144 assessed patients, 19 of them for incomplete clinical data; the application of adult WHO anemia thresholds to the 43 patients older than 65 years; the absence of anemia severity, etiology, and iron-status data; the absence of muscle strength and physical performance measures, which precludes a functional sarcopenia diagnosis; the pooling of post-treatment and primary pathological T and N categories; and the retention of 20 completion gastrectomies, which increases case-mix heterogeneity and adhesion-related operative difficulty. Larger multicenter observational cohorts should prespecify CT and laboratory timing, use validated population-appropriate thresholds together with muscle strength and performance, document measurement reliability, capture perioperative confounders and post-discharge events, and accrue enough outcomes for defensible adjustment.

## 5. Conclusions

In this consecutive, predominantly neoadjuvant-treated gastric-carcinoma cohort, preoperative CT-defined low skeletal muscle mass, impaired nutritional–immune status, and anemia were each common, affecting approximately two-thirds, two-fifths, and two-thirds of patients, respectively. Their relation to in-hospital major morbidity could not be resolved: with 15 events, every interval estimate remained compatible with both a clinically meaningful protective and a clinically meaningful harmful association; the estimates were unchanged by an alternative muscle threshold that altered exposure prevalence almost threefold whereas exclusion of completion gastrectomies reversed the direction of the dichotomous muscle estimate without improving its precision; and the concentration of events among patients undergoing open surgery suggests that operative case mix may have contributed substantially to the observed distribution of major morbidity events, although these data could neither confirm nor quantify that contribution. Because these abnormalities were common, it is reasonable to hypothesize that their systematic preoperative assessment could contribute to perioperative risk stratification; this hypothesis was not tested here, the present data cannot support it as a recommendation, and it should be evaluated in adequately powered prospective studies with prespecified adjustment for operative variables. From a methodological standpoint, cohorts of this size should report the width of their uncertainty rather than dichotomous test results, state the threshold and its derivation whenever body-composition prevalence is reported, and not present unadjusted estimates as independent predictors. Resolving the question will require multicenter cohorts with standardized CT and laboratory timing, population-appropriate thresholds, functional muscle assessment, documented measurement reliability, complete perioperative covariates, and outcome surveillance extending beyond index discharge.

## Figures and Tables

**Table 1:** Clinical, operative, tumor, body-composition, nutritional, and hematologic characteristics of the gastric-carcinoma cohort by major morbidity status.

Variable	Overall Cohort *n* = 107	No Major Morbidity *n* = 92	Major Morbidity *n* = 15
Age, years	62.4 ± 11.3	61.8 ± 11.1	66.3 ± 12.2
Female sex	29 (27.1)	23 (25.0)	6 (40.0)
BMI, kg/m^2^	25.0 [22.1–28.6]	25.4 [22.0–28.6]	24.8 [22.8–28.0]
ASA III–IV	50 (46.7)	42 (45.7)	8 (53.3)
Neoadjuvant therapy	86 (80.4)	74 (80.4)	12 (80.0)
Open surgical approach	76 (71.0)	62 (67.4)	14 (93.3)
Distal gastrectomy	30 (28.0)	23 (25.0)	7 (46.7)
Proximal gastrectomy	2 (1.9)	2 (2.2)	0 (0.0)
Total gastrectomy	55 (51.4)	51 (55.4)	4 (26.7)
Completion gastrectomy	20 (18.7)	16 (17.4)	4 (26.7)
Lymph nodes retrieved, *n*	22 [18.5–28.5]	22 [19.0–28.0]	22 [15.5–30.0]
Recorded T3–T4 category	54 (50.5)	44 (47.8)	10 (66.7)
Recorded N-positive category	56 (52.3)	48 (52.2)	8 (53.3)
Poorly differentiated/signet-ring carcinoma	56 (52.3)	45 (48.9)	11 (73.3)
Moderately differentiated carcinoma	37 (34.6)	34 (37.0)	3 (20.0)
Well-differentiated carcinoma	14 (13.1)	13 (14.1)	1 (6.7)
Skeletal muscle index, cm^2^/m^2^	46.5 ± 9.2	46.9 ± 9.4	44.0 ± 7.7
CT-defined low skeletal muscle mass (Prado)	67 (62.6; 95% CI 53.2–71.2)	57 (62.0)	10 (66.7)
CT-defined low skeletal muscle mass (Zhuang)	23 (21.5; 95% CI 14.8–30.2)	19 (20.7)	4 (26.7)
PNI	48.4 [42.4–52.3]	48.4 [43.0–52.2]	48.0 [41.8–51.7]
Low PNI (<46)	45 (42.1; 95% CI 33.1–51.5)	38 (41.3)	7 (46.7)
Hemoglobin, g/dL	11.9 ± 1.7	11.8 ± 1.8	12.3 ± 1.4
Anemia	71 (66.4; 95% CI 57.0–74.6)	61 (66.3)	10 (66.7)
C-reactive protein, mg/L	3.0 [1.0–10.0]	3.0 [1.0–8.5]	3.0 [2.0–16.5]

Data are mean ± SD, median [IQR], or *n* (%) with Wilson 95% CIs for the binary exposures in the overall cohort. Major morbidity was Clavien–Dindo grade ≥III during the index hospitalization. The table is descriptive; no significance testing was performed. ASA, American Society of Anesthesiologists; BMI, body mass index; CT, computed tomography; IQR, interquartile range; PNI, Prognostic Nutritional Index; SD, standard deviation.

**Table 2:** Exploratory unadjusted associations with in-hospital Clavien–Dindo grade ≥III morbidity in the gastric-carcinoma cohort.

Exposure	Events/Total	Conventional OR (95% Wald CI)	Wald *p* Value	Firth OR (95% Profile CI)	Firth PLR *p* Value
CT-defined low skeletal muscle mass	10/67	1.23 (0.39–3.89)	0.727	1.18 (0.40–3.84)	0.771
SMI, per 5-cm^2^/m^2^ decrease	15/107	1.20 (0.88–1.65)	0.255	1.19 (0.88–1.65)	0.266
PNI, per 5-unit decrease	15/107	1.18 (0.80–1.75)	0.411	1.18 (0.80–1.73)	0.396
Low PNI (<46)	7/45	1.24 (0.42–3.72)	0.697	1.25 (0.42–3.65)	0.683
Anemia	10/71	1.02 (0.32–3.23)	0.978	0.98 (0.33–3.20)	0.969

Conventional ORs were estimated via ordinary binary logistic regression with Wald 95% CIs and two-sided Wald *p* values. Firth ORs use Jeffreys-prior bias reduction, profile penalized-likelihood 95% CIs, and two-sided penalized likelihood-ratio *p* values. Each row is a separate one-exposure model. All estimates are unadjusted and exploratory and must be read in light of the concentration of 14 of the 15 events among open procedures. No multivariable, joint-exposure, interaction, or modified Poisson model was retained because 15 events did not support reliable estimation of those analyses. *p* values are tabulated for completeness only; interpretation in the text is based on the point estimates and interval widths. CI, confidence interval; OR, odds ratio; PNI, Prognostic Nutritional Index; SMI, skeletal muscle index.

**Table 3:** Postoperative outcomes and principal recorded major complications.

Postoperative Outcome	Value	Denominator/Summary
Length of stay, overall	9.0 [7.5–12.0] days	Median [IQR], *n* = 107
Length of stay, no major morbidity	8.5 [7.0–11.0] days	Median [IQR], *n* = 92
Length of stay, major morbidity	21.0 [13.0–34.5] days	Median [IQR], *n* = 15
Clavien–Dindo grade III	12 (80.0%)	Among 15 major events
Clavien–Dindo grade IV	2 (13.3%)	Among 15 major events
Clavien–Dindo grade V/in-hospital death	1 (6.7%)	Among 15 major events
Pancreatic fistula	4 (26.7%)	Principal complication
Bleeding	3 (20.0%)	Principal complication
Duodenal stump leak	3 (20.0%)	Principal complication
Duodenal stump and anastomotic leaks	1 (6.7%)	Principal complication
Evisceration	1 (6.7%)	Principal complication
Pneumothorax	1 (6.7%)	Principal complication
MODS	1 (6.7%)	Principal complication
Anastomotic stenosis	1 (6.7%)	Principal complication

For severity and complication type, percentages use the 15 patients with major morbidity as the denominator. The highest Clavien–Dindo grade per patient was retained. Each patient is represented once by the principal recorded complication; one patient had both a duodenal stump leak and an anastomotic leak. Events after index discharge were not captured. IQR, interquartile range; MODS, multiple organ dysfunction syndrome.

**Table 4:** Sensitivity analysis using alternative skeletal muscle index thresholds and excluding completion gastrectomies.

Analysis/Exposure	Dataset and Definition	Events/Exposed	OR (95% CI)	Firth OR (95% CI)
Low muscle mass—principal	107 patients, Prado thresholds	10/67	1.23 (0.39–3.89)	1.18 (0.40–3.84)
Low muscle mass—alternative threshold	107 patients, Zhuang thresholds	4/23	1.40 (0.40–4.88)	1.47 (0.40–4.67)
Low muscle mass—completion excluded	87 patients, Prado thresholds	6/53	0.74 (0.21–2.65)	0.73 (0.21–2.60)
Low muscle mass—alternative threshold, completion excluded	87 patients, Zhuang thresholds	3/17	1.66 (0.39–7.07)	1.77 (0.40–6.63)
SMI per 5-cm^2^/m^2^ decrease—completion excluded	87 patients	11/87	1.26 (0.86–1.84)	1.23 (0.87–1.84)
PNI per 5-unit decrease—completion excluded	87 patients	11/87	1.08 (0.67–1.75)	1.09 (0.67–1.72)
Low PNI (<46)—completion excluded	87 patients	5/35	1.28 (0.36–4.56)	1.29 (0.36–4.43)
Anemia—completion excluded	87 patients, WHO thresholds	7/59	0.81 (0.22–3.02)	0.78 (0.22–2.97)

The outcome is in-hospital Clavien–Dindo grade ≥III morbidity in every row. The 87 patient dataset excludes the 20 completion gastrectomies and contains 11 of the 15 events; for continuous exposures, the events/exposed column shows events/total. Zhuang thresholds were <40.8 cm^2^/m^2^ for men and <34.9 cm^2^/m^2^ for women; Prado thresholds were <52.4 and <38.5 cm^2^/m^2^. CI, confidence interval; OR, odds ratio; PNI, Prognostic Nutritional Index; SMI, skeletal muscle index; WHO, World Health Organization.

## Data Availability

The datasets used and/or analyzed during the current study are available from the corresponding author on reasonable request.

## Supplementary Materials

The following supporting information can be downloaded at https://www.mdpi.com/article/10.3390/medicina62101949/s1. Table S1: Sensitivity analysis reinstating the five records excluded on histological grounds (gastrointestinal stromal tumor).

## Abbreviations

The following abbreviations are used in this manuscript:

PNI	Prognostic Nutritional Index
CT	Computed Tomography
BMI	Body Mass Index
ASA	American Society of Anesthesiologists
WHO	World Health Organization
AJCC	American Joint Committee on Cancer
SMI	Skeletal Muscle Index
STROBE	Strengthening the Reporting of Observational Studies in Epidemiology
OR	Odds Ratio
CI	Confidence Interval
IQR	Interquartile Range
SD	Standard Deviation
GIST	Gastrointestinal Stromal Tumor
MODS	Multiple Organ Dysfunction Syndrome
