# Supplementary material for: CT-Defined Low Skeletal Muscle Mass, Prognostic Nutritional Index, and Anemia in Relation to In-Hospital Major Morbidity After Gastrectomy for Gastric Carcinoma: A Retrospective Cohort Study
Source: Medicina (Kaunas). 2026 Oct 9;62(10):1949. doi: 10.3390/medicina62101949 (PMC13706997; doi:10.3390/medicina62101949)
Supplement: Supplementary file 1 [file medicina-62-01949-s001.zip › medicina-4535465-supplementary.pdf]

Supplementary Materials

CT-Defined Low Skeletal Muscle Mass, Prognostic Nutritional Index and Anemia in Relation to In-Hospital Major Morbidity After Gastrectomy for Gastric Carcinoma: A Retrospective Cohort Study

Table S1. Sensitivity analysis reinstating the five records excluded on histological grounds (gastrointestinal stromal tumor): exploratory unadjusted associations with in-hospital Clavien–Dindo grade ≥III morbidity in the resulting 112-record dataset.

Exposure	Events/n	Prevalence, n (%; 95% CI)	OR (95% Wald CI)	Firth OR (95% profile CI)
Low skeletal muscle mass (Prado)	10/70	70 (62.5; 53.3–70.9)	1.23 (0.39–3.89)	1.18 (0.40–3.84)
SMI, per 5-cm²/m² decrease	15/112	—	1.22 (0.88–1.68)	1.20 (0.89–1.68)
PNI, per 5-unit decrease	15/112	—	1.17 (0.79–1.73)	1.17 (0.79–1.72)
Low PNI (<46)	7/48	48 (42.9; 34.1–52.1)	1.20 (0.40–3.56)	1.20 (0.41–3.50)
Anemia (WHO)	10/75	75 (67.0; 57.8–75.0)	0.98 (0.31–3.12)	0.95 (0.32–3.09)

The 112-record dataset contains the same 15 events as the 107-patient gastric-carcinoma cohort; no event occurred among the five reinstated records. Each row is a separate one-exposure model. Conventional ORs were estimated by ordinary binary logistic regression with Wald 95% CIs; Firth ORs use Jeffreys-prior bias reduction with profile penalized-likelihood 95% CIs. Prevalence intervals are Wilson 95% CIs. Low skeletal muscle mass was defined by the Prado thresholds (<52.4 cm²/m² for men, <38.5 cm²/m² for women); anemia by the 2024 WHO sex-specific thresholds. The analysis is exploratory and was not used to select a preferred estimate. CI, confidence interval; OR, odds ratio; PNI, Prognostic Nutritional Index; SMI, skeletal muscle index; WHO, World Health Organization.
